# Supplementary material for: Impact of left ventricular assist devices and heart transplants on acute myocardial infarction and heart failure mortality and readmission measures
Source: PLoS One. 2020 Mar 26;15(3):e0230734. doi: 10.1371/journal.pone.0230734 (PMC7098556; doi:10.1371/journal.pone.0230734)
Supplement: Attachment — Submitted filename: PLOS ONE Reviewer Responses.docx [file pone.0230734.s001.docx]

ONE-D-19-30581: Impact of left ventricular assist devices and heart transplant on acute myocardial infarction and heart failure mortality and readmission measures

Editor’s comments:

Thank you for submitting your manuscript to PLOS ONE. After careful consideration, we have decided that your manuscript does not meet our criteria for publication and must therefore be rejected. Specifically, there were concerns whether this study is novel or much additive to the knowledge in the field, while the conclusions were deemed vague and the clinical implication of these results questionable. I am sorry that we cannot be more positive on this occasion, but hope that you appreciate the reasons for this decision.

Response: We thank the editor for his consideration of our paper and that the reviewers were provided a timely response. However, we were confused as to why the decision was to reject. Our manuscript clearly meets the criteria for publication in PLOS ONE as outlined on the journal website. Most importantly we disagree with the assertion that the study is not novel or does not add to the field. This study has not been previously published nor the hypothesis tested previously. While the findings may be “expected,” our understanding was that part of the purpose of PLOS ONE was to publish research that is scientifically valid and meets methodological and ethical standards – not perceived significance. That said, the subject of our study, the impact of inclusion of LVAD and heart transplant patients on hospital measures of healthcare quality, has been a concern for key stakeholders and therefore represents an important and significant topic. We feel our study is both important and significant since they were the primary data that CMS used to make the decision of including or excluding these individuals from mortality and readmission measures. These measures remain the focus of national debate and continue to be key measures for hospital quality. Lastly, as had been pointed out by reviewers, our study was performed with high technical standards and are presented clearly. Therefore, since we feel our article meet all criteria and represent important findings we request an appeal for the decision to reject this manuscript.

Thank you for your reconsideration.

Reviewers' comments:

Reviewer #1: The authors conduct an interesting study examining the rate of risk standardized mortality rates and readmission rates in an acute myocardial infarction and heart failure population with respect to heart failure and LVAD patients.

1. Abstract comments: In results section would specify exact number, less than or equal to 0.2% does not make sense in this context. Same with less than or equal to 0.1%. How could the number of patients be less than or equal to? Shouldn’t this be a whole integer? Would refer to semi-permanent/permanent as the indication for the LVAD, either bridge-to-transplant (semi-permanent) or destination therapy (permanent)

Response: Thank you for these suggestions. We agree that it will help readers have a better understanding of the size of the cohort, as well as the number with devices, by providing this information in the abstract. In addition, we have considered the change in phrasing from semi-permanent/permanent to bridge-to-transplant or destination therapy. However, after discussion among the team, we respectively feel it is best to utilize the original terms. This will help to prevent misunderstanding regarding the intentions of the LVAD, since this cannot be necessarily construed from the indication. Nevertheless, in response to this comment, we have updated the abstract text to include the size of the cohort and number of devices in the abstract as follows (page 2): “In the AMI mortality (n=506,543) and readmission (n=526,309) cohorts, 1,166 and 1,016 patients received an LVAD while 3 and 2 received HT, respectively. In the HF mortality (n=1,015,335) and readmission (n=1,254,124) cohorts, 789 and 931 received and LVAD, while 212 and 202 received HT, respectively.”

2. Introduction: Drawing similarities between devices like CF-LVADs and percutaneous support devices is rather heterogeneous. The indications of these devices and devices are far too different to conflate them. May be better to state actual devices (Impella for percutaneous support, Heartmate II and III for durable devices). Later in the intro paragraph, these are again conflated. When CF-LVAD or LVAD is written, most people would default to an assumption of a durable CF-VAD. The authors should clarify the distinction between percutaneous and durable devices.

   Response: We agree with the reviewer that CF-LVADs and percutaneous support devices often have different indications. Percutaneous devices are much more likely to be used in acute care scenarios such as AMI whereas CF-LVADs are better suited for chronic scenarios such as chronic HF. Ideally we would have known the exact devices used in each scenario. However, these data were not available within the dataset since the administrative codes were not granular enough to identify a specific device. However, this limitation does not detract from the primary purpose of this study, which was to understand the impact of including patient that receive heart transplant or any LVAD type devices on mortality and readmission measures. Furthermore, we did use available data on device indication to guide interpretation of our analyses. The fact that LVADs were primarily indicated for acute care in AMI but for chronic care in HF suggests that, as this reviewer hints, percutaneous devices are more often employed in the former verse the latter scenario. We include these points within the discussion, which also responds to concerns from Reviewer #4 (page 16): “Notably, indication for LVAD use in the AMI and HF populations greatly differed between these two populations. This suggests that LVAD and HT are employed in disparate clinical scenarios and should be considered separately;” We have also recognized the specific limitation this reviewer points out (page 18): “Additionally, since data were not available as to type of LVAD utilized, comparisons could not be made between types of LVAD (i.e. centrifugal vs. axial vs. percutaneously inserted devices).”
3. Materials and methods: The inclusion of CF-LVAD and percutaneous support devices needs to be acknowledged in the limitations since they have different indications. This is partially acknowledged in the limitations section. Durable CF-LVAD patients and percutaneous support patients are not exactly similar.

   Response: The reviewer is correct in pointing out that these devices have distinct indications. As mentioned in the above response, we would ideally be able to separate out the indication and determine which exact device was implanted and in which exact clinical scenario. These data do not allow for this type of evaluation. As such we have expanded our limitations to address this issue. Please note that at other points in the text we are sure to point out the importance in type of LVAD and how that was important for deciding which devices to include or exclude in measures. We have update the text as follow, which also helps to address concerns from response #1 (page 18): “Furthermore, these data did not allow for a determination of whether the indication for use was semi-permanent or permanent, as in, whether devices were intended as bridge-to-transplant or destination therapy. Thus, evaluating our findings in the context of these clinical goals could not be discussed.”
4. For the heart transplant patients, these are patients who were transplanted at this index hospitalization for heart failure?

   Response: Yes, that is correct, patients in these respective cohorts received devices or transplant during the index hospitalization.
5. Discussion: Page 17, “supports their being difference” should be corrected to there

   Response: This has been correct in the text to read as follows (page 17): “…supports there being differences…”.
6. General comments: Can the authors clarify if these were pulsatile flow LVADs or continuous-flow? Given the contemporary cohort, it would be assumed that the LVAD population is continuous flow. Given that, the authors should replace LVAD at each instance with continuous-flow LVAD or CF-LVAD.

   Response: These data do not provide data on the level of understanding types of LVADs that were implanted beyond the indication. Given that the type of LVAD implanted is not adjudicated from these data we feel it would be inaccurate to make this assumption that they were all continuous flow LVADs. We have pointed this out in the manuscript with the following sentence of the limitations (page 18): “Additionally, since data were not available as to type of LVAD utilized, comparisons could not be made between types of LVAD (i.e. centrifugal vs. axial vs. percutaneously inserted devices).”
7. Did this population include patients who had RVAD devices placed? Were any total artificial hearts included in the cohort?

   Response part 1: The ICD-9CM code specifying RVAD fall under 37.66, which were included in our cohort definition. We expect based on clinical practice that these were a minority of the population sampled. To reflect this limitation, we have included a statement in the limitations (page 18): “Furthermore, ICD-9CM code 37.66 also includes right ventricular assist devices, thus there were likely some individuals with these devices included in the cohort. Although, these are clinically less common and were likely rare events.”

   Response part 2: This population did not include patients with total artificial hearts placed. Text was added to the methods and limitations to clarify this point (page 5): “This population did not include patients with total artificial hearts.” See also (page 18): “This study also did not include the ICD-9CM code for total artificial hearts (37.52).”
8. Limitation: by including only Medicare patients a significant number of patients are not included in the cohort some recent studies of 200 patients cite a mean age of 59. CF-LVAD patients have both a destination therapy and bridge-to-transplant indication. Did this study include both patient populations?

   Response: The reviewer is correct that our study was limited to Medicare patients, but not individuals with commercial or other types of insurance. As such, the reviewer is correct that if the mean age of LVAD implantation is below the typical Medicare age then majority of the patients for which this applies were not included in the study. However, because the purpose of our study was to understand the impact of including these patients on the measures used by Medicare for purposes of characterizing hospital quality of care (which are limited to patients enrolled in fee-for-service Medicare), there would be no reason to include patients who did not have Medicare insurance. Nevertheless, to be responsive to the Reviewer’s concern, we have updated our manuscript to reflect that this study was limited to only include Medicare patients and therefore was not inclusive of the total population (page 18): “Also, our study was limited to Medicare patients only and did not include patients that have commercial insurance only. Results should be interpreted in this context.”

Reviewer #3:

1. In this retrospective analysis of Medicare free-for-service beneficiaries with primary diagnosis of acute myocardial infarction (AMI) or heart failure (HF), Brandt et al presented the effects of including LVAD and heart transplant (HT) patients on 30-day readmission and mortality measures. The authors found the including or excluding these patients of patients does not significantly impact these measures though there were more readmission and less mortality among patients implanted an LVAD or underwent HT. The authors then concluded that LVAD and HT patients are still a distinct group with different care requirements and outcomes and should be analyzed separately from other groups of patients with HF/AMI. The manuscript is well written and methods are appropriate. However, these findings are largely expected and not novel. The clinical implication is questionable as the number of patients with HT or LVAD is very small relative to the large group of patients with chronic HF or AMI.

   Response: We thank the reviewer for his complement that our manuscript is well written with appropriate methods. However, we disagree with the assertion that the findings are not novel and lack implications. First, these findings have not been previously published in the literature and, as such, are novel. Second, this study has direct policy relevance, which in turn gives it great clinical relevance, since it was used by CMS to decide on inclusion and exclusion criteria for the mortality and readmission measures used by the agency to characterize hospital quality of care. Adding these findings to the literature provides scientific merit to the decisions as well as adds the scientific discourse on how these measures are determined.

Reviewer #4:

1. The authors of “Impact of left ventricular assist devices and heart transplant on acute myocardial infarction and heart failure mortality and readmission measures” speak to whether or not exclusion of LVAD and heart transplant patients significantly change the outcomes of CMS public reporting readmission and mortality for acute MI and heart failure patients. The statistics are done rigorously, and the manuscript is well-written in easy to understand English.

   Response: We thanks this reviewer for the complement
2. The authors utilized modeling similar to past publications to calculate the risk standardized readmission rate and mortality rate for these hospitals, and ultimately compared whether or not these values changed when LVAD and HT patients were excluded from the cohort. In the abstract, the authors state that their data supports that HT and LVAD patients should be considered separately from the all-comer HF patient population, although the fact that the exclusions do not change the RSMR and RSRR does not support that conclusion. Furthermore, the data show a similar lack of change in RSMR and RSRR in the AMI, which the authors do not discuss. In the discussion of the paper, the authors state that there is a dichotomy in AMI patients vs HF patients, in that the indication for LVAD shows that the AMI patients had external LVADs placed, vs HF patients receiving durable LVAD options, and that this likely drove the decision for the CMS to exclude HT and LVAD patients from their HF data, but not AMI data. In the discussion the authors do not really state whether or not they believe that HT and LVAD patients should be excluded from the HF and AMI groups, they simply discuss what the CMS policy reflects. Overall this discussion is confusing because the abstract and discussion of the paper do not deliver a consistent message, it would be helpful for the reader if the authors discussed why they believe their findings do/do not support the CMS policy decisions.

   Response: We appreciate the Reviewer’s comment that our abstract and discussion text do not align in tone. We have looked closely at both sections of our manuscript and revised our language to ensure that it is clear that CMS is the ultimate decision maker of how to utilize the data for policy, as well as our own interpretations of the research, independent of CMS. As such, we have updated the discussion to parallel the tone of the abstract while still emphasizing CMS as the primary decision maker for defining inclusion criteria for mortality and readmission measures. We have updated the text as follows, which also touches on concerns from Reviewer #1 (page 16): “Notably, indication for LVAD use in the AMI and HF populations greatly differed between these two populations. This suggests that LVAD and HT are employed in disparate clinical scenarios and should be considered separately. The data we present here were the primary considerations in the CMS decision to exclude LVAD and heart transplant patients from HF, but not AMI, mortality and readmission measures.”
3. Furthermore, it is hard to believe that the exclusion of the LVAD patients and HT patients would affect the RSMR and RSRR values for the entire cohort, these exclusions make up ~1,000/1,000,000 of the HF patients (1/1000th of the cohort) and 1/500th of the AMI group. This is not discussed by the authors, it seems as though it is statistically impossible for the exclusion of such a small group to meaningfully shift the entire cohort’s mortality/morbidity.

   Response: In regards to this observation, many stakeholders had noted concerns that despite making up a small portion of the population that they could have significant implications for individual hospitals. We did confirm that they are a small portion of the population but did not confirm concerns that they significantly alter performance. Notably we did bring attention to the small proportion of patients with LVAD and HT that make up the cohort. We were sure to point out that these patients make up a small portion of the cohort throughout the manuscript.

Some points for consideration:

4. What was the purpose of stratifying LVADs into three groups and HT in to two groups? A discussion of this would be helpful to the reader

   Response: The purpose of the stratification scheme for LVADs was to see whether higher volume centers would be more affected. Due to the number of transplant being low we could only do two groups and not three.
5. Table 3B has incredibly small sample size for hospitals with more than 1 transplant. All data regarding heart transplant on AMI patients is based on an incredibly small sample size. It is unlikely that these data hold up across all hospitals in the united states or abroad considering that there are only three hospitals that are doing transplant in this cohort. Also, the clinical track to receiving a transplant in the setting of an acute MI is not entirely clear.

   Response: The reviewer is correct in pointing out that heart transplant as a result from AMI hospitalization is a rare event. *A priori* this could have been predicted. However, these data are important, despite the small counts, for considering populations to be included or excluded from admission and readmission measures. As mentioned above in response #3, this was important to for stakeholders to understand whether these patients alter performance measures.
6. In table 3a, 3b, 4a and 4b Why are the N values for hospitals with hospitals with and without HT/LVAD patients different in terms of mortality and readmissions? Shouldn’t the N value for these hospitals be equal in terms of hospitals that had heart failure patients, that were considered for readmission/mortality data? Were hospitals that have no deaths and no readmissions excluded from the reported N? For example, there is a discrepancy of over 100 hospitals between mortality and readmission hospitals with no transplants.

   Response: The CMS inclusion and exclusion criteria used to define the mortality and readmission measures for the cohorts have differences. This results in slightly different values for hospitals that are included in the individual cohorts. Hospitals that had no patients meeting criteria were not included in the study cohorts. However, if a hospital had patients meeting criteria but these patients had no readmission or deaths then these were included.
7. The authors state that “Hospitals caring for >6 patients with LVAD or >1 HT had lower RSMRs but higher RSRRs on average.” It would be helpful to know what confidence intervals there are for this methodology. Most of the differences are less than 1% (one is equal, in fact), are we confident the modeling can say that these are truly different?

   Response: The term on average could be construed as a statistical term. However, in this context we are referring to non-statistical comparisons of medians. As such we have corrected the text for the readers to understand that in these conclusions. We have updated the text remove the term “on average” and it now reads as follows in 2 locations (page 3): “Hospitals caring for ≥6 patients with LVAD or ≥1 HT typically had lower RSMRs but higher RSRRs.” (page 19): “In conclusion we report that hospitals caring for patients with LVAD or performed heart transplants typically had lower RSMR but higher RSRR, which were not significantly changed when these patients were excluded.”
8. It is worth adding a discussion regarding the shortcomings of using the database from 2010 to 2013. This data is being derived from the previous generation of LVADs (at least from a durable LVAD perspective). The authors should acknowledge that all of the data presented in this paper regarding devices are not commonly being used at this time (Impellas have been redesigned, Heartmate 3, and HVAD have been approved since the study period). Overall, it is not clear why the authors chose to use the CMS data from 2010-2013, when they feasibly could have used 2013-2016 (up until the date that the CMS changed their reporting guidelines). It would be helpful to know why the authors chose these dates; at face value it does not seem like these are the most recent data available to the authors. The fact that all most LVADs that are used today are a newer generation from what is being studied in this paper should be discussed by the authors.

   Response part 2: It is noted that pointing out that newer devices may have differing outcomes. It is also worth noting that this is an important piece to continued efforts to study mortality and readmissions in relation to transplant and LVADs. The text has been updated to include the following text (page 18): “Notably, the data were from 2010-2013, which reflects outcomes from an older generation of LVAD devices. More recent data were not available to the authors for the purposes of this study.”
