# Supplementary material for: Impact of left ventricular assist devices and heart transplants on acute myocardial infarction and heart failure mortality and readmission measures
Source: PLoS One. 2020 Mar 26;15(3):e0230734. doi: 10.1371/journal.pone.0230734 (PMC7098556; doi:10.1371/journal.pone.0230734)
Supplement: Attachment — Submitted filename: PLOS ONE reviewer comments_2.docx [file pone.0230734.s002.docx]

**Additional Editor Comments (if provided):**

1. The authors intended to assess the impact of LVAD and HTx, a subgroup, on some outcome measures. These surgical cases were a small minority and stat. insig changed those rates. In epidemiology, it is always better to enhance homogeneity to reduce selection bias. So it was not a bad idea to exclude those cases. But since their existence did not affect the rates significantly by statistical testing, why not just keep them to reflect the reality in clinical scenario? The authors needed to clarify the purpose of those outcome measures. Is there any benefit to exclude LVAD and HTx, such as for cost management, admin, insurance, etc? Please revise to reflect the motivation of the manuscript. LVAD and HTx, as a small minority, did not affect the outcome measures. Their non-exclusion did not violate homogeneity but also reflect the diversity of clinical reality. The authors need to provide a stronger reason to exclude them

   Response: We appreciate the Editor’s questioning whether it was appropriate to exclude this small minority of patients, since inclusion in the models did not materially affect the hospital risk-standardized rates. The portion of the cohort that had LVAD and HTx were predictably small. However, our *a priori* methodology decision to exclude them and repeat the analysis was essential to addressing and being responsive to stakeholder and policymaker concerns, Providing reassurance that managing these patients does not lead to changes in hospital mortality and readmission since these measures could alter reimbursement and potentially patient care. We have made this argument more clear by addition to the introduction (page 5): “Exclusion of LVAD and heart transplant patients will inform whether these patients significantly alter hospital level mortality and readmissions, thus informing stakeholder and policymakers on the implications of having them included or excluded from future measures.”

**Comments to the Author**

Reviewer #5: PONE-D-19-30581_R1

The authors of “Impact of left ventricular assist devices and heart transplantation on acute myocardial infarction and heart failure mortality and readmission measures” sought to understand the frequency of deaths and readmissions in patients supported on advanced heart failure therapies as a component of Medicare population-based metrics to determine if inclusion of such patients unduly affects the Medicare-based assessment of those hospitals performance with respect to those measures. Certainly, these data are important to have available for consideration, but the way in which some of the data and conclusions are presented could be misleading and in the interest of disseminating this information, authors should seek to clarify this for potential authors who would cite these data.

Major Comments:

1. What was the authors’ intent with including temporary/external LVADs? I would suggest that the authors completely exclude external or temporary LVADs (and why do the authors indicate in the limitations that they could not distinguish between percutaneous LVADs vs. durable VADs?). The authors ultimately argue that LVAD patients should be considered separately, but I am unsure if patients supported on temporary LVADs should be excluded given the controversy of using these devices (i.e. Impella) routinely. Protected PCI may be another indication during AMI admission for use of an “LVAD” which further complicates the data. Additionally, the controversy with respect to the HRRP primarily surrounds the HF admission indication and the observation of the disparate mortality and readmission data (as the authors also observed) perhaps unduly affecting high quality centers. I believe these data are of much more relevance wherein the proportion of durable LVADs is much higher.

   Response: We thank the reviewer for the thoughtful discourse about our work. The reviewer recognizes the importance of our manuscript, that the populations for which LVADs are used have different indications for use as well as different clinical scenarios. This is precisely why our data were important for CMS and why temporary/external LVADs were important to include. We found that LVADs used in the AMI cohort more often had indications for temporary rather than chronic support needs. The data do not inform the exact type of LVAD nor whether the devices were semi-permanent or permanent. This limitation of the data cannot be overcome, nor are there additional data available that would refine our ability to differentiate the exact device type. Excluding temporary/external LVADs from the data would significantly alter the relevance of our findings, a portion of which were to inform on decisions to include or exclude LVAD patients from the AMI cohort. The majority of the devices in the HF cohort were implanted device. Even when including patients with external/temporary LVADs, which would bias us away from our current conclusions, the data still support excluding these patients. Thus, we expect our findings are more robust by continuing to include these patients.
2. However if the authors cannot readily separate temporary and durable device and so choose to proceed with inclusion of temporary LVADS, in the first methods paragraph, I would provide examples of the external or temporary LVAD versus semi-permanent or permanent LVADs for clarity for the reader to emphasize throughout the idea that markedly different LVAD devices with markedly different indications are lumped into his cohort.

   Response: As discussed above, we cannot readily separate these devices. We have edited the text to provide examples of devices available at the time of the study. Page 4: “Some devices are delivered percutaneously for temporary hemodynamic support (i.e. Impella® and TandemHeart® devices at the time of the study), while others are implanted for intermediate to longer term support as destination therapy or bridge to transplant (i.e. HeartMate® II, at the time of our study).”
3. Previous reviewers have criticized the manuscript for the statistical inability to generate differences between cohorts after removing LVAD and transplant patients. While I agree with the essence of these comments, I disagree about the resultant lack of importance of the manuscript. The manuscript, as I interpret it, is designed to provide a neutral assessment of the data in the CMS calculations. In that regard, the authors have done well in not applying a statistical test of significance. However, I would clarify that objective within the introduction and discussion. For example, rather than stating in the introduction that they “sought to examine the impact of …” I would suggest that the authors indicate that they sought to quantify the available data on LVAD and HTx mortality and readmissions as it relates to the CMS decision. Similarly, in the limitations, in the limitations, I would suggest that the authors indicate that due to very small numbers of patients in the cohorts of interest in a large administrative database, the study was not powered to detect differences and thus no statistical tests of significance were used.

   Response: We appreciate the Reviewer’s comment and recognize that we had not made this point clear in our prior version of our manuscript. We have edited the manuscript with the language suggested (page 7): “Due to the small numbers of patients with LVAD and heart transplantation in the cohorts, the study was not powered to detect differences and thus no statistical tests of significance were used.”

Minor comment:

4. In the 3rd to last sentence of the first Methods paragraph, I believe the authors mean HTx cohort rather than HF cohort.

   Response: The reviewer is correct and we had made an error in this sentence. We have corrected this error, see page 5: “Inclusion in the cohorts was defined by ICD-9CM procedure codes 37.60, 37.62, 37.65. 37.66. and 37.68 for the LVAD cohort and 33.6 and 37.51 in the heart transplant cohort.”

Reviewer #6: Brandt et al. have evaluated mortality and readmission measures of acute myocardial infarction and heart failure patients while assessing the impact of including patients receiving LVAD and heart transplants on these measures. The authors provide a revision of their manuscript along with responses to prior reviewer comments. The manuscript is well-written and presented in an intelligent fashion. The statistical methodology appears adequate although further description of the same in the methods section would be ideal. The authors have addressed most of the concerns raised in a reasonable manner. Most importantly it is apparent that they are limited by the data available to them with respect to providing granularity on this topic. Although I am inclined towards the author’s suggestion that these are important findings given their importance in policy making, I do have a few comments for the authors’ consideration,

1. Firstly, the choice of using data from 2010 to 2013 is surprising. The authors have addressed this concern in response to prior comments citing availability of data as an issue. However, as previously pointed out most devices part of this study are no longer in use and as such do not reflect current clinical practice. Given the authors primary objective was to provide basis for the CMS decision to exclude LVAD and heart transplant patients, I would urge them to include data up until 2016 as suggested earlier.

   Response: We agree that using the most available data is always best, when possible. The reviewer points out that the devices used today differ from that during our study period. However, the frequency of device use has also changed since the period from 2014 through 2016. Thus, we don’t think that adding data through 2016 would reflect on current device utilization either since the most frequently utilized implanted LVAD (the Heartmate III) was not approved by the FDA for use until 2017. Lastly, the decision to exclude LVAD and heart transplant patients was based on the data presented in our manuscript and not data from after 2013. Thus, we feel it makes the most sense to present on the data that were utilized in this decision. Finally, but also critically, data from after 2013 are not currently available to the authors. Obtaining agreement to analyze post-2013 would require additional contracting and is not feasible.
2. I am not entirely convinced with the conclusion that “Hospitals caring for >6 patients with LVAD or >1 HT typically had lower RSMRs but higher RSRRs” given the differences are less than 1% and the small cohort sizes (LVAD and heart transplant groups). Although the authors suggest that they are referring to this in a non-statistical manner, I would suggest saying “Hospitals caring for >6 patients with LVAD or >1 HT typically had a trend toward lower RSMRs but higher RSRRs”.

   Response: We agree with the reviewer that the altered statement more accurately reflects the data. Thus, we have updated the manuscript accordingly in both the abstract and the conclusion. See pages 3 and 19, which now read as follows: “Hospitals caring for ≥6 patients with LVAD or ≥1 patients with heart transplantation typically had a trend toward lower RSMR but higher RSRR.” and “…hospitals caring for patients with LVAD or performed heart transplants typically had a trend toward lower RSMR but higher RSRR,” respectively.
3. The authors acknowledge that their findings are expected and reiterate the importance of the findings to stake holders and decision makers. However, given the limitations of the database, the unavailability of data on types of LVAD, indications for use and the small number of patients receiving LVAD and heart transplant, it is hard to establish clinical precedence for this manuscript. Having said that, I would certainly urge the editor to consider this for publication provided the authors are able to add data up until 2016 when CMS made the policy change to provide more comprehensive findings representing that decision.

   Response: We thank the reviewer for recognizing the importance of our findings for stake holders and decision makers. Please see our complete response above as to why it is not feasible to include data through 2016.
